# Quantitative facial analysis using emotrics in patients undergoing vestibular schwannoma surgery

**DOI:** 10.1007/s00701-026-06933-0

**Published:** 2026-06-04

**Authors:** Adéla Bubeníková, Zdeněk Fík, Michaela Tesařová, Lenka Peterková, Vladimír Koucký, Jan Lazák, Anna Kabo, Amir Gorchakov, Jan Betka, Eduard Zvěřina, Ondřej Bradáč, Aleš Vlasák

**Affiliations:** 1 Department of Neurosurgery, 2nd Faculty of Medicine, Charles University in Prague and Motol University Hospital, Prague, Czech Republic; 2 Department of Otorhinolaryngology and Head and Neck Surgery, 1st Faculty of Medicine, Charles University in Prague and Motol University Hospital, V Úvalu 84, Prague, 150 06 Czech Republic

**Keywords:** Vestibular schwannoma, Facial asymmetry, Facial nerve, Facial recovery, Vestibular schwannoma surgery

## Abstract

**Objective:**

Facial nerve dysfunction is a common complication after vestibular schwannoma (VS) resection. Traditional grading scales such as House-Brackmann (HB), Sunnybrook, and Fisch are subjective and prone to inter-rater variability. Emotrics, a computer vision–based tool, offers objective facial analysis, but its role in neurosurgical populations remains underexplored.

**Methods:**

Patients undergoing VS surgery were prospectively enrolled in this study. Standardized frontal facial photographs were taken preoperatively, at discharge, and at three-month follow-up. Emotrics quantified facial symmetry and movement across static, dynamic, and synkinesis domains. Composite scores were generated and compared with clinical grading scales and patient characteristics.

**Results:**

Thirty-six patients (mean age 51.2 ± 12.3 years) were included. Emotrics detected significant improvement in dynamic function (mean score: 0.41 at discharge vs. 0.56 at 3 months, *p* < 0.01) and synkinesis (0.62 to 0.51, *p* < 0.05); static symmetry remained stable. Strongest correlations with clinical grading were found at discharge and 3 months. Discharge Emotrics total scores correlated significantly with HB (*r* = –0.883, *p* < 0.001), Sunnybrook (*r* = 0.892, *p* < 0.001), and Fisch (*r* = 0.883, *p* < 0.001). At 3 months, dynamic scores remained strongly associated with HB (*r* = –0.799), Sunnybrook and Fisch (*r* = 0.790; *p* = 0.001). Worse Emotrics outcomes were linked to older age (62.1 vs. 48.6 years, *p* = 0.04) and larger tumor volume (8.2 vs. 4.5 cm^3^, *p* = 0.03).

**Conclusions:**

Emotrics offers objective assessment of facial nerve function, correlates well with clinical scales, and enhances evaluation of dynamic facial recovery. Its integration may refine postoperative monitoring and guide rehabilitation.

**Supplementary Information:**

The online version contains supplementary material available at 10.1007/s00701-026-06933-0.

## Introduction

Facial nerve dysfunction remains one of the most functionally limiting complications following vestibular schwannoma (VS) resection, often resulting in varying degrees of facial paralysis that adversely affect patients’ functional capabilities, aesthetic appearance, and psychosocial well-being [15, 19]. Preservation and accurate assessment of facial nerve function are therefore pivotal components of VS surgical management and postoperative care. Current standard clinical assessment methods rely primarily on grading scales such as House-Brackmann, Sunnybrook, and Fisch classifications [12, 21]. While these scales provide a framework for evaluating facial nerve integrity, they are inherently subjective and susceptible to significant inter- and intra-observer variability as well as lower sensitivity to detecting surgically facilitated recovery [12]. Such variability is further compounded by differences in examiner expertise, limiting the sensitivity and reproducibility of clinical assessments, particularly in subtle or evolving facial nerve dysfunction [20].

Recent advances in computer vision and machine learning have introduced digital tools that facilitate objective, quantitative analysis of facial movement and symmetry [7]. Emotrics is a computer vision–based tool that automatically extracts quantitative facial metrics and has been proposed as the basis for standardized electronic facial grading systems. In the present study, we use Emotrics-derived facial symmetry and movement indices as an objective modality and compare them with established clinical facial nerve grading scales [3, 13]. The software quantifies parameters such as palpebral fissure height, oral commissure displacement, and nasolabial fold symmetry, thereby enabling detailed, reproducible assessment of facial nerve function. Initial studies of Emotrics in patients with facial nerve palsy secondary to various etiologies have demonstrated favorable reliability and validity when compared with established clinical scales [4, 8]. However, to date and to our best knowledge, there is no data validating Emotrics specifically within the VS patient population, in whom facial nerve impairment may present distinct temporal and functional patterns due to tumor characteristics, surgical approach, and postoperative recovery dynamics.

Moreover, demographic factors such as patient age and sex, as well as tumor laterality, may influence baseline facial symmetry and the trajectory of postoperative facial nerve recovery [17], yet their impact on objective facial metrics derived from Emotrics remains largely unexplored. The establishment of objective, sensitive, and reproducible assessment tools is critical not only for enhancing clinical decision-making and personalized rehabilitation strategies but also for facilitating multicenter research and clinical trials through standardized outcome measures [20].

Within this context, the present study was undertaken to evaluate the potential applicability and reliability of Emotrics, an automated computer vision–based modality for objective facial nerve assessment, in patients undergoing surgical treatment for VS. The study aims to contribute evidence toward the role of objective digital facial analysis tools in the clinical assessment and longitudinal monitoring of facial nerve function in VS patients.

## Methods

Patients diagnosed with vestibular schwannoma and indicated for surgical resection were prospectively enrolled. Standardized frontal facial photographs were acquired at three timepoints: prior to surgery (baseline), before home discharge a week after surgery, and at three-month follow-up. Facial function was quantified using Emotrics software (Massachusetts Eye and Ear Infirmary, Boston, MA, USA) [9], which provides quantitative landmark-based measures and composite scores. The eFACE score was not collected separately in this cohort. Subjective clinical assessment was performed independently by eight blinded raters using the House–Brackmann, Sunnybrook, and Fisch grading systems.

**Fig. 1:**
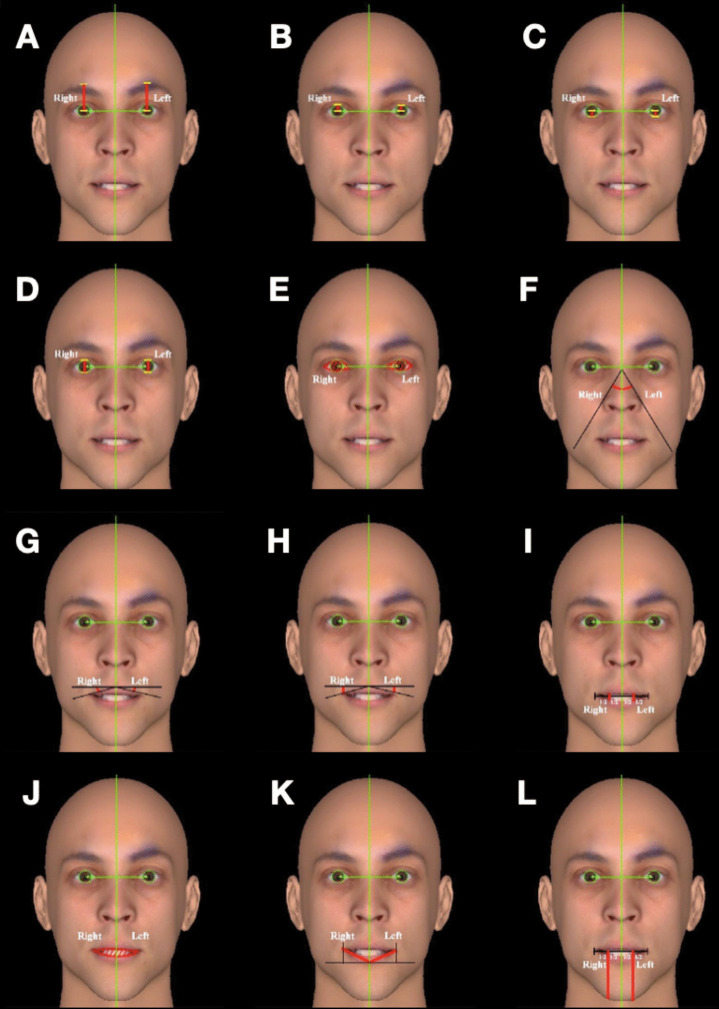
Emotrics metric-based facial measurements description. **A** Brow height (mm); **B** Marginal reflex distance 1 (mm), i.e. the vertical distance from the green line to the upper eyelid margin; **C** Marginal reflex distance 2 (mm), i.e. the vertical distance from the green line to the lower eyelid margin; **D** Palpebral fissure width (mm); **E** Eye area (mm^2^); **F** Nasolabial fold angle (degree); **G** Upper lip slope (degree); **H** Commisure height with respect to upper lip vermilion border (mm); **I** Interlabial distance (mm); **J** Interlabial area of the hemiface (mm.^2^); **K** Commisure position with respect to midline lower lip (mm); **L** Lower lip height (mm)

Emotrics-derived facial metrics were obtained using automated landmark detection of periorbital, perinasal, and perioral regions. Facial photographs were acquired with a single, tripod-mounted camera (Olympus E-P2 – M.Zuiko Digital 14–42 mm) in a dedicated room with daylight excluded, using a uniform background and constant indoor lighting; the camera was aligned at eye level, perpendicular to the facial plane, and kept at a fixed distance of 2.0 m from the patient for all sessions. For each region of interest, Emotrics quantifies absolute position at rest (static symmetry) and displacement during standardized voluntary movements (dynamic excursion). In general, for rest parameters (e.g., brow position at rest, palpebral fissure height at rest, oral commissure position at rest, nasolabial fold depth), values closer to inter-side symmetry indicate better facial nerve function; increasing asymmetry indicates impaired resting tone or static deviation. For dynamic parameters (e.g., brow raise, smile excursion, lower lip movement, eye closure), larger and more symmetrical excursion is interpreted as better voluntary motor output of the facial nerve. Synkinesis metrics capture involuntary co-contraction in non-target regions during voluntary movement; higher synkinesis values therefore indicate worse pathological co-activation. Composite scores are interpreted as follows: higher Static and Dynamic Scores indicate more normal/restored symmetry and excursion, whereas higher Synkinesis Score reflects more severe involuntary co-contraction. The Total Score is an integrated index of overall facial function, with higher values corresponding to globally better facial symmetry, movement, and control (Table 1, Fig. 1). All measurements were obtained for both the affected and unaffected sides to quantify asymmetry.

**Table 1:** Emotrics parameters and descriptions

Parameter	Description	Interpretation of values
Score Brow Rest	Horizontal position of the eyebrow at rest, indicating static brow asymmetry	Values closer to left–right symmetry reflect preserved resting frontalis tone and intact facial nerve function; larger deviations indicate static asymmetry or brow ptosis on the weaker side
Score Palpebral Fissure Rest	Vertical distance between upper and lower eyelids at rest	More symmetric palpebral fissure height between sides indicates normal resting orbicularis oculi tone; marked inter-side difference suggests lagophthalmos or eyelid retraction on the affected side
Score Oral Commissure Rest	Horizontal position of the oral commissure at rest, reflecting facial symmetry	Values approaching midline symmetry reflect preserved resting perioral tone; inferior/lateral droop or horizontal displacement of one commissure indicates static lower facial paresis
Nasolabial Fold (NLF) at Rest	Depth and presence of the nasolabial fold, a marker of resting tone	A well-formed, symmetric nasolabial fold is consistent with preserved zygomatic/buccal branch function; flattening or loss of the fold on one side indicates hypotonia of midfacial mimetic musculature
Brow Raise	Superior movement of the eyebrow during maximal frontalis activation	Greater and more symmetric superior excursion indicates better voluntary activation of the frontalis; reduced or asymmetric excursion indicates impaired dynamic upper facial function
Gentle Eye Closure	Degree of eyelid approximation during light eye closure	Complete and symmetric gentle closure suggests preserved orbicularis oculi function; incomplete or asymmetric gentle closure suggests mild facial weakness
Full Eye Closure	Completeness of eyelid closure during forceful contraction	Full, symmetric occlusion of the palpebral fissure indicates intact forceful orbicularis function; residual palpebral gap or marked asymmetry indicates clinically relevant upper facial palsy
Oral Commissure with Smile	Degree of upward and lateral movement of the mouth corner during smile	Larger and more symmetric superolateral excursion of the oral commissure corresponds to better dynamic lower facial function; blunted or asymmetric excursion indicates paresis of zygomatic/buccal branches
Lower Lip Movement	Vertical and horizontal displacement of the lower lip during movement	Larger symmetric displacement reflects preserved marginal mandibular branch output; reduced excursion or deviation toward the healthy side suggests lower facial weakness
Ocular Synkinesis	Involuntary eyelid movement during voluntary facial actions (e.g., smiling)	Higher values indicate more pronounced pathologic co-contraction (synkinesis), i.e. abnormal orbicularis oculi activation during non-ocular tasks; lower values indicate better selective motor control
Static Score	Composite score summarizing asymmetry at rest across facial zones	Higher Static Score indicates more normal resting symmetry and tone (better function); lower Static Score indicates greater static asymmetry
Dynamic Score	Composite score summarizing symmetry during active facial expressions	Higher Dynamic Score indicates more symmetric and complete voluntary excursion across facial regions (better dynamic facial nerve function); lower Dynamic Score indicates impaired movement
Synkinesis Score	Composite score reflecting the presence and severity of involuntary movements	Higher Synkinesis Score indicates more severe unwanted co-contraction (worse synkinesis); lower Synkinesis Score indicates better selective activation
Total Score	Overall facial function score derived from static, dynamic, and synkinesis components	Higher Total Score represents globally better facial symmetry, movement, and motor selectivity. Lower Total Score reflects more severe weakness and/or synkinesis

Clinical facial nerve grading was independently performed by eight raters: two medical students, two neurosurgical residents, two board-certified consultants, and two senior attending physicians each performing measurements in a blinded fashion to evaluate intra-rater reliability using the House-Brackmann (HB), Sunnybrook, and Fisch grading systems at each timepoint [12, 21]. Demographic variables, including patient age, sex, and lesion side (left/right), Koos grade, and intra-/extracanalicular extension (INT) were recorded for subgroup analyses. Tumor size was measured in accordance with the AAO-HNS guidelines [1].

## Data analysis

Continuous variables were reported as mean ± standard deviation or median with interquartile range, depending on the distribution assessed via the Shapiro–Wilk test. Longitudinal changes in Emotrics-derived facial function parameters were evaluated using repeated-measures analysis of variance (ANOVA) for normally distributed data, and the Friedman test for non-normally distributed data. Post-hoc comparisons were performed using Tukey’s HSD or Wilcoxon signed-rank tests, as appropriate.

To assess the consistency of facial nerve grading across raters, inter-rater and intra-rater reliability were calculated using intraclass correlation coefficients (ICCs). Inter-rater reliability was estimated using a two-way random-effects model with absolute agreement (ICC(2,1)).

Associations between automated Emotrics scores and clinical grading scales (HB, Sunnybrook, and Fisch) were examined using Pearson or Spearman correlation coefficients, depending on data distribution.

Subgroup analyses explored the effects of demographic and clinical variables (e.g., age, sex, tumor volume, lesion laterality) on facial function outcomes using correlation analysis and Mann–Whitney U or t-tests, as appropriate. A *p*-value < 0.05 was considered statistically significant. All statistical analyses were performed in an open-source R environment (version 4.4.3).

## Results

### Patient demographics and baseline characteristics

A total of 36 patients diagnosed with only sporadic VS were included in the study. The cohort consisted of 64% females and 36% males. The mean age at the time of surgery was 51.2 years (SD ± 12.3). Tumor volume had a mean of 7.4 cm^3^ (SD ± 9.6). Most patients were operated via retrosigmoid approach (94.4%), only two were operated via the translabyrinthine approach (5.6%). According to the Koos classification, most tumors were grade 4 (55.56%). The median INT was 2, ranging from 0 to 5. Regarding the surgical outcome, gross total resection was reached in 86.1% of patients, the remaining were near-total. More demographic information is presented in Table 2.

**Table 2:** Cohort summary

Parameter	Value
Number of patients	36
Sex	
Female	23/36 (63.9%)
Male	13/36 (36.1%)
Age (years)	Mean 51.2 ± 12.3
Tumor side	Left: 20/36 (55.6%), Right: 16/36 (44.4%)
Koos grade	4 (IQR 2–4)
INT grade	2 (IQR 1–3)
Extent of resection	
Gross-Total	31/36 (86.1%)
Near-Total	5/36 (13.9%)
Tumor size (cm^3^)	Median 6.23 (IQR 1.21–18.69)
House–Brackmann	
Preop	Median: 1.0 (IQR 1.0–1.0)
Postop	Median: 2.0 (IQR 1.0–4.0)
3 months	Median: 1.0 (IQR 1.0–4.0)
Sunnybrook	
Preop	Median: 100.0 (IQR 100–100)
Postop	Median: 88.0 (IQR 34–100)
3 months	Median: 100.0 (IQR 86–100)
Fisch	
Preop	Median: 100.0 (IQR 100–100)
Postop	Median: 88.0 (IQR 38–100)
3 months	Median: 100.0 (IQR 73–100)

### Automated facial function assessment using emotrics

**Fig. 2:**
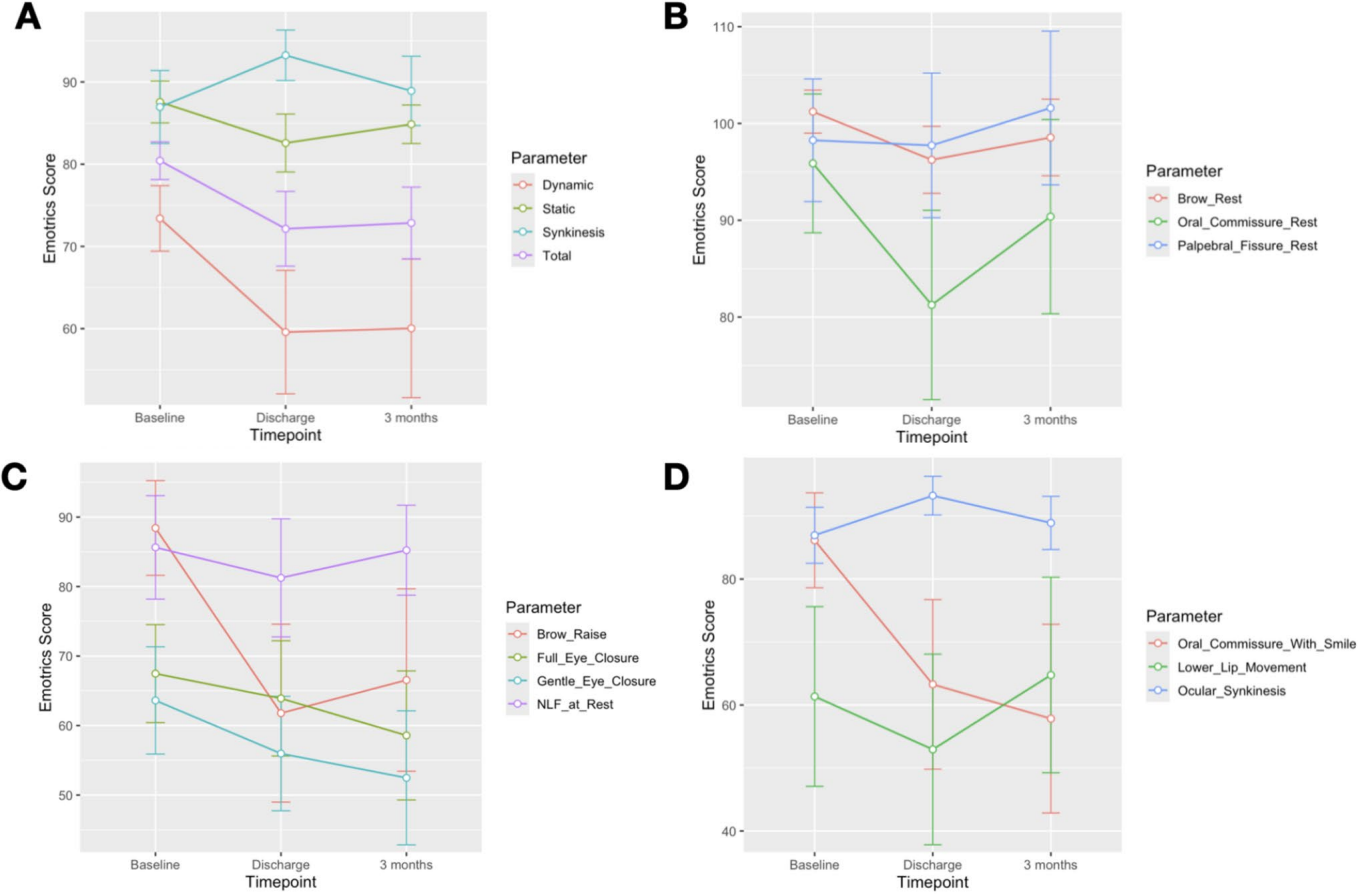
Longitudinal analysis of changes across timepoints in the studied Emotrics variables (A-D; the list of used parameters written on the right). White circles represent mean values, while vertical bars indicate ± SD, reflecting variability within each group. Colored lines show trends over time for each domain

Longitudinal analysis using the Friedman test revealed significant changes across timepoints in several facial domains, including Brow Rest (χ^2^ = 7.0, *p* = 0.030), Lower Lip Movement (χ^2^ = 7.6, *p* = 0.022), and Ocular Synkinesis (χ^2^ = 7.6, *p* = 0.022) (Fig. 2). No significant temporal changes were detected in Palpebral Fissure Rest, Oral Commissure Rest, Nasolabial Fold at Rest, Brow Raise, Gentle Eye Closure, or Full Eye Closure. These findings indicate selective regional improvements during facial nerve recovery, particularly involving lower facial musculature and synkinetic patterns.

### Trends in static, dynamic, and synkinesis summary scores

Repeated-measures ANOVA demonstrated significant temporal effects on the Dynamic (F = 4.02, *p* = 0.022) and Total (F = 4.28, *p* = 0.017) Emotrics scores (Fig. 2). The Synkinesis domain showed a trend toward significance (F = 3.10, *p* = 0.051), whereas changes in the Static score did not reach statistical significance (F = 2.12, *p* = 0.127). These results were corroborated by Friedman tests, which confirmed significant improvement in the Synkinesis score over time (χ^2^ = 9.79, *p* = 0.007). Post-hoc comparisons indicated that the majority of changes occurred between baseline and the 3-month follow-up. Collectively, these results suggest a measurable recovery in facial mobility and global symmetry, particularly in dynamic movements.

### Comparison between clinical and automated grading

**Fig. 3:**
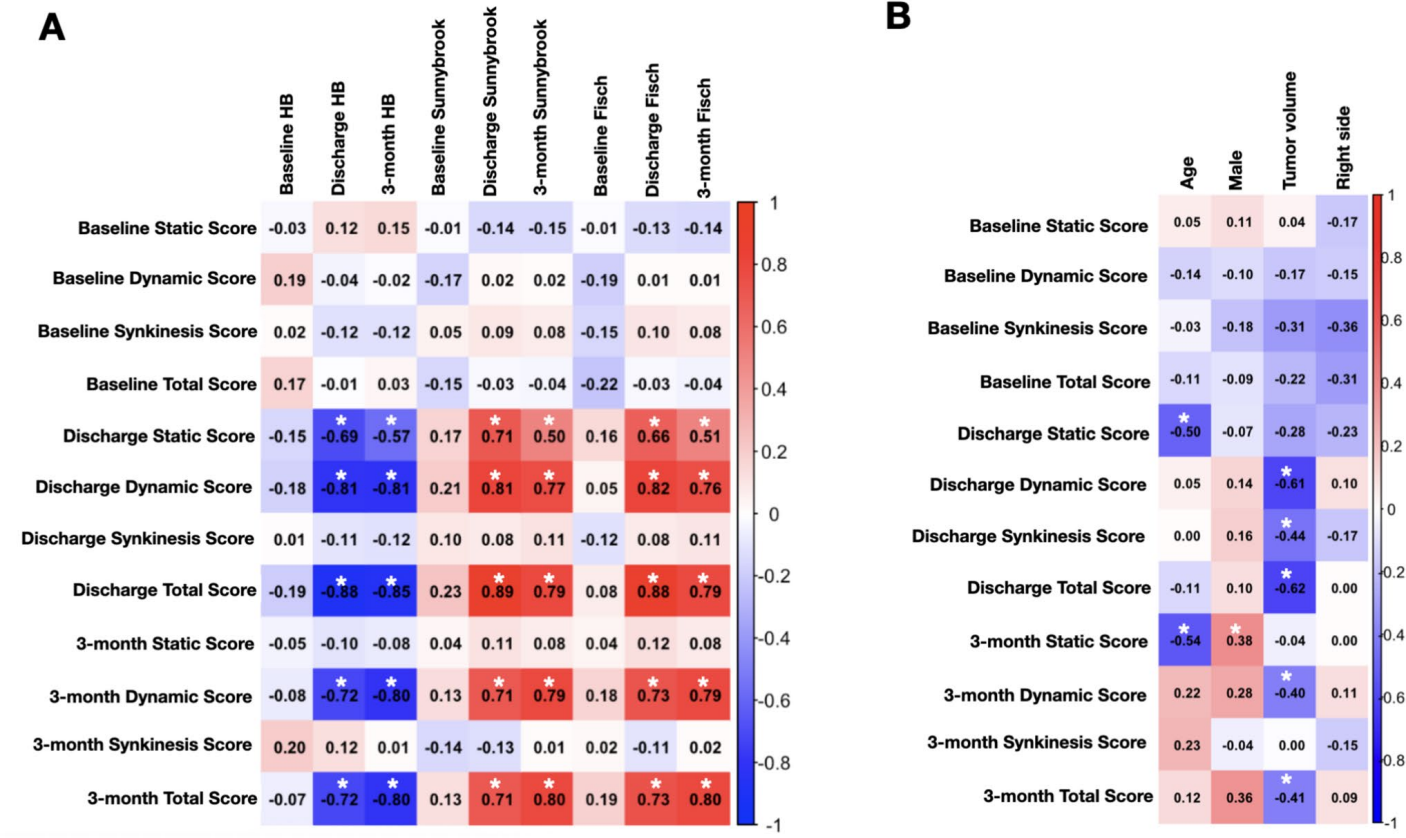
Heatmap of correlation between emotrics scores and clinical scores at baseline, discharge, 3-month follow-up. Emotrics score (A) and demographic variables (B). White asterix indicates statistically significant result *p* < 0.05. Note: In HB, lower scores indicate better function, while in SB and Fisch, higher scores are better. Color direction may differ, but clinical meaning is comparable. **B** Heatmap of correlation between Emotrics scores and demographic variables. White asterix indicates statistically significant result *p* < 0.05

Correlation analysis revealed meaningful associations between facial function scores and clinical grading systems (HB, Sunnybrook, and Fisch) across multiple time points (Fig. 3).

The most robust associations were observed at discharge and 3 months postoperatively. At discharge, Emotrics dynamic scores exhibited strong negative correlations with HB (*r* = –0.81, *p* < 0.001) and strong positive correlations with both Sunnybrook (*r* = 0.81, *p* < 0.001) and Fisch scores (*r* = 0.82, *p* < 0.001). Total Emotrics scores at this time point showed even stronger correlations with HB (*r* = –0.88, *p* < 0.001), Sunnybrook (*r* = 0.89, *p* < 0.001), and Fisch (*r* = 0.88, *p* < 0.001).

At the 3-month follow-up, these relationships persisted. Emotrics dynamic scores remained significantly correlated with HB (*r* = –0.80, *p* < 0.001), Sunnybrook (*r* = 0.79, *p* < 0.001), and Fisch (*r* = 0.79, *p* < 0.001). Likewise, total Emotrics scores correlated with HB (*r* = –0.80), Sunnybrook (*r* = 0.80), and Fisch (*r* = 0.80), all at *p* < 0.001.

### Subgroup analyses by demographic variables

A correlation analysis (Fig. 3) was conducted to explore associations between facial function scores at different timepoints (baseline, discharge, and 3-month follow-up) and demographic variables including age, gender, affected side, tumor volume, and preservation of the cochlear nerve after surgery. Notably, significant negative correlations were found between 3-month Total scores and tumor volume (*r* = –0.407, *p* = 0.035), as well as between discharge Total score and tumor volume (*r* = –0.617, *p* = 0.001). In addition, older age was associated with lower Static scores at discharge (*r* = –0.504, *p* = 0.012) and at 3 months (*r* = –0.541, *p* = 0.009). A significant positive correlation was observed between male gender and 3-month Static score (*r* = 0.380, *p* = 0.048).

### Temporal changes in clinical facial nerve grading

Friedman analysis of HB scores showed a significant effect across time (χ^2^ = 46.67, *p* < 0.001), with post-hoc tests indicating worse function from baseline to discharge (*p* < 0.001), improvement from discharge to 3 months (*p* < 0.01), and no significant difference between baseline and 3 months—consistent with transient postoperative decline followed by recovery (Figure presented in Supplementary Fig. 1).

Sunnybrook scores likewise differed across time on one-way ANOVA (F = 11.26, *p* < 0.001), decreasing from baseline to discharge (*p* < 0.001) and improving from discharge to 3 months (*p* < 0.001), with baseline vs 3 months not significant (*p* = 0.600), suggesting return to baseline function (Supplementary Fig. 2).

A significant decrease was observed in Fisch scores between the preoperative and discharge assessments (*p* < 0.001), and between discharge and 3-month follow-up (*p* < 0.001). A smaller but still statistically significant difference was also found between preoperative and 3-month scores (*p* < 0.001). These results indicate that facial nerve function, as assessed by the Fisch score, significantly declined after surgery but improved again by the 3-month follow-up, though not fully to baseline values.

### Interrater reliability of clinical grading systems

The interrater reliability of three facial nerve grading systems (HB, Sunnybrook, and Fisch) was evaluated across three timepoints: baseline, discharge, and 3-month follow-up. Baseline agreement was poor–moderate (ICC 0.143–0.405), highest for Sunnybrook (ICC 0.405, 95% CI 0.277–0.561). These low ICCs likely reflect minimal variability at diagnosis (most patients HB I), where homogeneity reduces the informativeness of ICC despite good clinical concordance. Reliability improved markedly postoperatively and at 3 months, peaking with Fisch at 3 months (ICC 0.988, 95% CI 0.980–0.994), followed by Sunnybrook (0.987) and HB (0.938). Improvements from baseline to later timepoints were significant for all scales (*p* < 0.001), whereas differences between discharge and 3 months were not, indicating consistently high agreement during follow-up (Table 3).

**Table 3:** Intraclass correlation coefficients for rater agreement at baseline, discharge, and 3-month follow-up

Scale	Comparison	ICC1	ICC2	Z	*p*-value
HB	Baseline vs Discharge	0.143	0.931	−13.49	< 0.001
HB	Baseline vs 3-months	0.143	0.938	−15.02	< 0.001
HB	Discharge vs 3-months	0.931	0.938	−0.48	0.628
Sunnybrook	Baseline vs Discharge	0.405	0.969	−17.34	< 0.001
Sunnybrook	Baseline vs 3-months	0.405	0.987	−19.78	< 0.001
Sunnybrook	Discharge vs 3-months	0.969	0.987	−3.99	< 0.001
Fisch	Baseline vs Discharge	0.168	0.979	−22.26	< 0.001
Fisch	Baseline vs 3-months	0.168	0.988	−24.44	< 0.001
Fisch	Discharge vs 3-months	0.979	0.988	−2.76	0.006

## Discussion

### Longitudinal recovery patterns captured by emotrics

This study demonstrates the feasibility and clinical relevance of using automated facial analysis via Emotrics to track facial nerve recovery following VS surgery. The longitudinal evaluation revealed domain-specific recovery trajectories. Notably, Dynamic and Total Emotrics scores improved significantly over time, particularly between baseline and the 3-month follow-up (*p* = 0.022 and *p* = 0.017, respectively), indicating enhanced voluntary movement and overall facial performance. Similarly, Synkinesis scores showed significant variation across timepoints (Friedman χ^2^ = 9.79, *p* = 0.007), suggesting an improvement in motor control and partial resolution of involuntary movements. In contrast, Static scores did not demonstrate significant changes over time, potentially reflecting early plateauing of resting facial symmetry or limited sensitivity of this parameter to subtle functional shifts.

These findings suggest that dynamic and synkinetic movements are more responsive markers of functional recovery, while static asymmetries may either recover earlier or exhibit minimal variability during follow-up. Therefore, dynamic facial performance and synkinesis may be more informative in both clinical assessment and therapeutic monitoring and should be prioritized in future outcome evaluations and intervention planning. In this context, although Emotrics provides standardized and reproducible metrics from static images, facial nerve function is inherently dynamic. Static photographs offer only a snapshot—typically of posed expressions—and may miss subtle deficits such as delayed activation, impaired coordination, or synkinetic movements. Video-based assessments, on the other hand, capture the full temporal evolution of facial motion, enabling a more comprehensive evaluation of functional performance [11]. Furthermore, video allows for frame-by-frame analysis, enhancing sensitivity to clinical changes and supporting more nuanced interpretation of recovery trajectories. As such, future tools that incorporate automated video analysis may offer superior clinical relevance, particularly when tracking progress over time or evaluating treatment response [11].

### Complementarity and limitations of clinical grading scales

Traditional clinical grading systems (HB, Sunnybrook, and Fisch) remain widely used in practice [6, 12, 22]. However, our results highlight their limitations in objectivity and inter-rater consistency, particularly at baseline. Preoperative inter-rater reliability was poor to moderate for all three systems (ICC range: 0.143–0.405), likely reflecting the challenge of detecting subtle deficits when facial function appears grossly intact. In contrast, inter-rater reliability improved significantly in the postoperative and 3-month assessments, where deficits were more pronounced and consistent across patients.

This temporal pattern suggests that clinical rating becomes more reproducible when facial impairment is more visible, while subtle asymmetries remain prone to subjective interpretation [16, 20]. These limitations underscore the need for adjunctive tools that can offer quantitative and reproducible assessments, especially in early recovery stages or in borderline cases.

### Value of emotrics as an objective assessment tool

In this context, Emotrics offers a valuable complement to clinical grading [3, 13]. Its ability to capture region-specific, quantitative, and automated facial metrics reduces observer bias and enhances reproducibility [20]. Our data demonstrate that Emotrics not only tracked functional recovery over time but also correlated well with clinical scores, especially during the postoperative and follow-up phases, supporting its validity as an outcome measure.

Importantly, correlations between Emotrics scores and clinical grading systems were weaker at baseline, likely due to reduced variability in facial function early after diagnosis. This suggests that Emotrics is sensitive to functional deterioration and recovery, aligning well with clinical evaluation when deficits are apparent, while avoiding overinterpretation when changes are minimal [14].

Our findings support and extend previous work validating the Emotrics software as a reliable tool for quantitative facial analysis [13, 20]. Kim et al. [13] demonstrated that Emotrics provides excellent intra- and inter-rater reliability (ICC 0.61–0.99) and significant correlations with established clinical grading systems such as HB, confirming its utility for objective facial function assessment. While their focus was primarily on cross-sectional validation and technical reproducibility, our study highlights the importance of temporal dynamics in interpreting Emotrics scores. Specifically, we observed that correlations between Emotrics and clinical scales were weaker at baseline, likely due to low variability in facial function shortly after diagnosis. In contrast, stronger correlations emerged during the postoperative and follow-up periods, suggesting that Emotrics is particularly sensitive to functional deterioration and recovery when clinically meaningful changes are present. Analogously, Mato-Patino et al. [18] showed that the eFACE had a very strong correlation with the HB (*p* < 0.001) and the Sunnybrook (*p* < 0.001) in their cohort of 65 adult patients with unilateral facial paralysis.

This temporal sensitivity underlines a key distinction: unlike clinical scores where absolute values often guide decision-making, the clinical utility of Emotrics lies in its ability to detect directional changes over time. Thus, it is not the absolute Emotrics values that are most informative, but rather their trajectory. This nuance is essential when using Emotrics in clinical research or longitudinal patient monitoring, where automated, unbiased tracking of facial dynamics can provide added value beyond traditional grading systems [4, 5].

### Implications for clinical practice and future research

The integration of eFace using Emotrics software into facial nerve assessment could standardize evaluations across centers and clinicians, reducing inter-rater variability and improving follow-up comparability. Its automated nature and high reproducibility also make it well suited for clinical trials and longitudinal patient monitoring.

In our exploratory subgroup analysis, several patient- and tumor-related variables were associated with Emotrics-derived facial function outcomes. Tumor volume showed a consistent negative correlation with Total Emotrics scores at both discharge and 3-month follow-up, supporting the relevance of lesion size for postoperative facial nerve function. Similarly, older age was associated with lower Static scores, consistent with prior evidence suggesting reduced regenerative potential with increasing age [2]. Male gender was positively associated with higher 3-month Static scores. While this finding should be interpreted cautiously given the limited cohort size and exploratory nature of the subgroup analysis, it may reflect subtle differences in resting facial symmetry, soft tissue characteristics, or recovery dynamics that are not captured by global clinical grading scales. These associations should therefore be regarded as hypothesis-generating but clinically relevant observations that merit validation in larger prospective studies.

Interestingly, male gender was positively correlated with higher 3-month Static scores, suggesting better facial symmetry at rest in men. This finding did not parallel HB scores, which showed no significant gender differences. One possible explanation is that Emotrics, by providing fine-grained, region-specific analysis, is more sensitive to subtle differences in facial symmetry that may not influence the global HB grade [10]. It is also possible that men in this cohort recovered static symmetry more rapidly due to differences in facial soft tissue tone, muscle mass, or behavioral use of facial muscles during rehabilitation. However, given the relatively small sample size and the exploratory nature of this analysis, these observations should be interpreted cautiously and validated in larger, prospective studies.

From a prognostic standpoint, dynamic Emotrics scores may serve as early markers of functional recovery potential, while persistent synkinesis could guide targeted rehabilitation [5]. Furthermore, Emotrics may help stratify patients for early intervention, assess response to physiotherapy or neuromodulation, and provide objective endpoints in surgical outcome studies.

Beyond objective monitoring, quantitative facial analysis may also contribute to future predictive models of facial nerve recovery after VS surgery. In the present study, Emotrics was used as a longitudinal assessment tool rather than as a validated prognostic classifier. Early postoperative Emotrics-derived measures, particularly Dynamic and Total scores, may capture subtle residual motor function and early recovery trajectories that are not fully reflected by conventional clinical grading scales. However, established normative thresholds and minimum clinically important differences for Emotrics composite scores are currently lacking. Therefore, although these scores are useful for quantifying within-patient longitudinal change and correlate with established clinical grading systems, the clinical meaning of a specific absolute Emotrics value remains uncertain. Until validated thresholds are available, Emotrics should be interpreted as a complementary objective assessment tool rather than as a standalone basis for rehabilitation or surgical decision-making. In future larger cohorts, Emotrics-derived parameters could be integrated with established prognostic factors such as age, tumor volume, preoperative facial function, surgical approach, extent of resection, intraoperative nerve monitoring, and early postoperative HB grade to develop models predicting facial nerve recovery at later follow-up. Such predictive use will require prospective multicenter validation, development of normalite thresholds, standardized imaging protocols, and external testing before clinical implementation.

### Limitations

Despite its potential, the use of Emotrics is not without limitations. Emotrics is publicly available as research software and can be used without major licensing costs in its publicly available form. In practice, the workflow is relatively straightforward. After standardized frontal photographs are uploaded, the software automatically detects facial landmarks and generates quantitative measurements of facial symmetry, movement, and synkinesis. In our experience, processing of one patient image usually requires only a few minutes. However, the process is not fully independent of user oversight. Image quality must be checked, and automatically detected landmarks should be visually inspected, with manual correction if needed. Therefore, although Emotrics substantially reduces the subjectivity and time burden of facial nerve assessment, reliable use still depends on standardized photography, basic familiarity with the software, and quality control of landmark detection.

First, the software relies on high-quality, standardized frontal facial photographs; variations in lighting, head position, and facial expression can introduce measurement errors and limit reproducibility across settings without strict imaging protocols. Second, Emotrics currently does not account for dynamic facial movements such as speech or spontaneous expression, which may limit its sensitivity to certain functional deficits [3]. Third, while it provides objective geometric data, the clinical interpretation of these metrics—particularly in terms of patient-reported function or psychosocial impact—remains to be fully validated. Additionally, the software’s performance in patients with facial asymmetries due to congenital conditions, trauma, or reconstructive procedures may differ from typical facial palsy cases, requiring further investigation.

In our series, although the dataset captures a range of facial nerve function, the relative under-representation of the most severe grades constrains the dynamic range available for validation at the lower end severity spectrum. Moreover, race, ethnicity, and skin tone were not prospectively recorded in this cohort, and we were therefore unable to assess whether these variables influenced Emotrics performance. Although image acquisition was standardized with fixed camera distance, uniform background, and constant indoor lighting, automated landmark-based analysis may still be affected by image contrast, shadows, facial hair, skin tone, and other photographic variables. Future validation studies should include racially and ethnically diverse cohorts and should specifically evaluate the performance of Emotrics across different skin tones, ideally using standardized reporting such as Fitzpatrick skin type or objective image-quality metrics.

Finally, while Emotrics provides reproducible landmark-based indices of resting symmetry, dynamic excursion, and synkinesis, the composite Static, Dynamic, Synkinesis, and Total Scores have not yet undergone prospective clinical validation in VS patients. Our results should therefore be considered preliminary and hypothesis-generating, requiring external confirmation. It also offers valuable trend data over time, but it should be viewed as a complementary tool rather than a replacement for clinician judgment and comprehensive clinical examination.

## Conclusion

Emotrics provides an objective and reproducible method for assessing facial nerve function, with particular sensitivity to dynamic deficits and synkinesis. In contrast to subjective clinical grading, which has limited inter-rater reliability, Emotrics enables consistent and detailed evaluation. These findings support ongoing efforts to develop an objective software tool for standardized facial function monitoring and outcome assessment in patients undergoing VS surgery. Emotrics should currently be considered a complementary objective assessment tool, while its potential role as part of a predictive model for facial nerve recovery remains an important direction for future research.

## Supplementary Information

Below is the link to the electronic supplementary material.

ESM 1 Supplementary Material 1: Clinical scoring of changes in facial function over time, namely graphs for Sunnybrook and Fisch classifications. (PNG 71.3 KB)

ESM 2 Supplementary Material 2: Clinical scoring of changes in facial function over time, namely graphs for HB score. (PNG 73.7 KB)

## Data Availability

No datasets were generated or analysed during the current study.
