# Supplementary figures and images for: Quantitative facial analysis using emotrics in patients undergoing vestibular schwannoma surgery
Source: Acta Neurochir (Wien). 2026 Jun 4;168(1):181. doi: 10.1007/s00701-026-06933-0 (PMC13427948; doi:10.1007/s00701-026-06933-0)

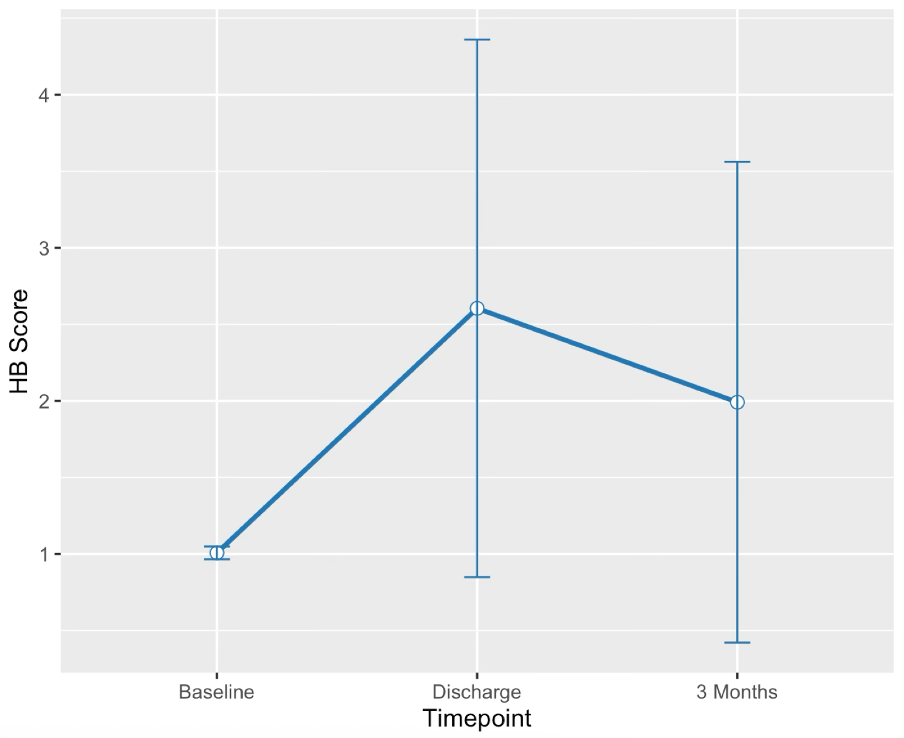

Supplement: Supplementary file 1 — Supplementary Material 1: Clinical scoring of changes in facial function over time, namely graphs for Sunnybrook and Fisch classifications. (PNG 71.3 KB) [file 701_2026_6933_MOESM1_ESM.png]

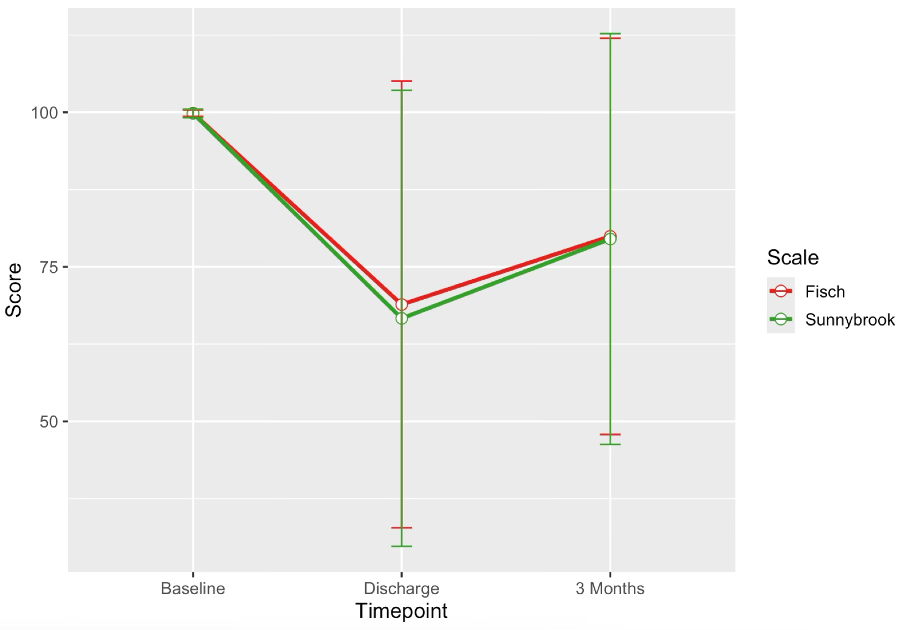

Supplement: Supplementary file 2 — Supplementary Material 2: Clinical scoring of changes in facial function over time, namely graphs for HB score. (PNG 73.7 KB) [file 701_2026_6933_MOESM2_ESM.png]
